# Vertical sleeve gastrectomy reduces blood pressure and hypothalamic endoplasmic reticulum stress in mice

**DOI:** 10.1242/dmm.027474

**Published:** 2017-03-01

**Authors:** Anne K. McGavigan, Zachariah M. Henseler, Darline Garibay, Scott D. Butler, Sisitha Jayasinghe, Ruth E. Ley, Robin L. Davisson, Bethany P. Cummings

**Affiliations:** 1 Department of Biomedical Sciences, College of Veterinary Medicine, Cornell University, Ithaca, NY 14853, USA; 2 Department of Molecular Biology and Genetics, Cornell University, Ithaca, NY 14853, USA; 3 Department of Microbiome Science, Max Planck Institute for Developmental Biology, 72076 Tübingen, Germany; 4 Cell and Developmental Biology, Weill Cornell Medical College, New York, NY 10065, USA

**Keywords:** Vertical sleeve gastrectomy, Hypertension, ER stress, Mouse model

## Abstract

Bariatric surgery, such as vertical sleeve gastrectomy (VSG), causes remarkable improvements in cardiometabolic health, including hypertension remission. However, the mechanisms responsible remain undefined and poorly studied. Therefore, we developed and validated the first murine model of VSG that recapitulates the blood pressure-lowering effect of VSG using gold-standard radiotelemetry technology. We used this model to investigate several potential mechanisms, including body mass, brain endoplasmic reticulum (ER) stress signaling and brain inflammatory signaling, which are all critical contributors to the pathogenesis of obesity-associated hypertension. Mice fed on a high-fat diet underwent sham or VSG surgery and radiotelemeter implantation. Sham mice were fed *ad libitum* or were food restricted to match their body mass to VSG-operated mice to determine the role of body mass in the ability of VSG to lower blood pressure. Blood pressure was then measured in freely moving unstressed mice by radiotelemetry. VSG decreased energy intake, body mass and fat mass. Mean arterial blood pressure (MAP) was reduced in VSG-operated mice compared with both sham-operated groups. VSG-induced reductions in MAP were accompanied by a body mass-independent decrease in hypothalamic ER stress, hypothalamic inflammation and sympathetic nervous system tone. Assessment of gut microbial populations revealed VSG-induced increases in the relative abundance of Gammaproteobacteria and *Enterococcus*, and decreases in *Adlercreutzia*. These results suggest that VSG reduces blood pressure, but this is only partly due to the reduction in body weight. VSG-induced reductions in blood pressure may be driven by a decrease in hypothalamic ER stress and inflammatory signaling, and shifts in gut microbial populations.

## Abstract (precis)

**Summary:** Vertical sleeve gastrectomy in mice decreases blood pressure independent of body mass, which may be due to a decrease in hypothalamic ER stress.

## INTRODUCTION

Bariatric surgery is currently the most effective long-term treatment for obesity and results in high rates of remission for type 2 diabetes and hypertension (Buchwald et al., 2004; Flores et al., 2014; Tritsch et al., 2015). Furthermore, bariatric surgery produces a greater reduction in arterial blood pressure (AP) than medical management alone (Halperin et al., 2014). However, the mechanisms by which this occurs remain elusive. While the mechanisms mediating the improvement in glucose homeostasis after bariatric surgery are being studied extensively, surprisingly few preclinical studies have been conducted to improve our understanding of how bariatric surgery causes hypertension remission.

Vertical sleeve gastrectomy (VSG) has gained popularity as a bariatric procedure that produces weight loss and improves glucose regulation and cardiovascular disease outcomes at rates similar to Roux-en-Y gastric bypass (RYGB) and is currently one of the most commonly performed bariatric procedures (Benaiges et al., 2011). VSG involves removal of ∼70% of the stomach by transecting the greater curvature of the stomach. In an effort to improve our understanding of the mechanisms driving the cardiometabolic benefits of bariatric surgery we have validated a mouse model of VSG (McGavigan et al., 2015). Consistent with observations in human patients, our murine VSG model exhibits decreased food intake and body mass, and body mass-independent improvements in glucose regulation (McGavigan et al., 2015). Therefore, we used our murine VSG model to develop and validate a mouse model that recapitulates the effect of VSG to reduce AP in humans. We then used this model to investigate potential mechanisms by which VSG surgery reduces AP.

Obesity is considered a major risk factor for the development of hypertension. Therefore, loss of body mass after bariatric surgery probably contributes to reduced AP. However, remission of type 2 diabetes and hypertension have been reported within days after surgery, prior to significant body mass loss (Ahmed et al., 2009; Pories et al., 1995). Thus, the human clinical literature suggests that there are body mass-independent mechanisms contributing to hypertension remission after bariatric surgery.

Two potential contributors to the body mass-independent effect of bariatric surgery to reduce AP are reductions in brain ER-stress signaling and shifts in gut microbial populations. ER-stress signaling is elevated in obesity and plays an integral role in the development of obesity-associated hypertension (Ozcan et al., 2004; Purkayastha et al., 2011b; Young et al., 2012). ER stress occurs when the folding capacity of the ER is exceeded and misfolded proteins accumulate leading to initiation of the unfolded protein response (UPR). The UPR is triggered by transmembrane sensors that detect unfolded proteins in the ER. These proteins are: PKR-like ER kinase (PERK), inositol-requiring enzyme 1α (IRE1α) and activating transcription factor 6 (ATF6) (Ron and Walter, 2007). The UPR is a highly evolutionarily conserved series of signaling pathways that act to restore ER homeostasis. For example, activation of PERK results in phosphorylation and inactivation of eukaryotic translation inhibition factor 2α (eIF2α), leading to decreased protein synthesis (Ron and Walter, 2007). Work in rodent models demonstrates that enhanced ER-stress signaling in brain areas, such as the hypothalamus, increases AP (Purkayastha et al., 2011b). Conversely, restoration of brain ER homeostasis markedly reduces AP (Young et al., 2012).

Alterations in gut microbial populations have been implicated in the pathogenesis of various cardiometabolic diseases, including hypertension (Turnbaugh et al., 2006; Yang et al., 2015). Specifically, obese and lean phenotypes are transmissible via inoculation of germ-free mice with obese or lean microbiomes (Turnbaugh et al., 2006). Similarly, a hypertensive phenotype is transmissible via inoculation of normotensive rats with a hypertensive microbiome (Durgan et al., 2016). Furthermore, previous work reports that transplantation of gut microbiota derived from RYGB-operated mice or humans to gnotobiotic mice produces metabolic improvements compared with control transplants (Liou et al., 2013; Tremaroli et al., 2015). Therefore, shifts in the gut microbiota may contribute to the cardiometabolic benefits of bariatric surgery. Although shifts in gut microbe populations after RYGB have been well-defined, few studies have investigated the impact of VSG on gut microbiota.

Identification of the mechanisms by which bariatric surgery reduces AP may reveal novel therapeutic strategies for hypertension treatment. Validation of an appropriate preclinical model is critical to take advantage of this opportunity to improve the care of patients suffering from hypertension. Here, we validate the first murine bariatric model that exhibits reductions in AP measured by gold-standard radiotelemetry technology. We demonstrate that this effect occurs, in part, independent of body mass. Finally, we present novel data showing that VSG-induced reductions in AP are associated with concurrent reductions in hypothalamic ER stress and inflammation, and shifts in gut microbial populations.

## RESULTS

### VSG surgery decreases energy intake, body mass and circulating leptin and ghrelin concentrations

**Fig. 1.**
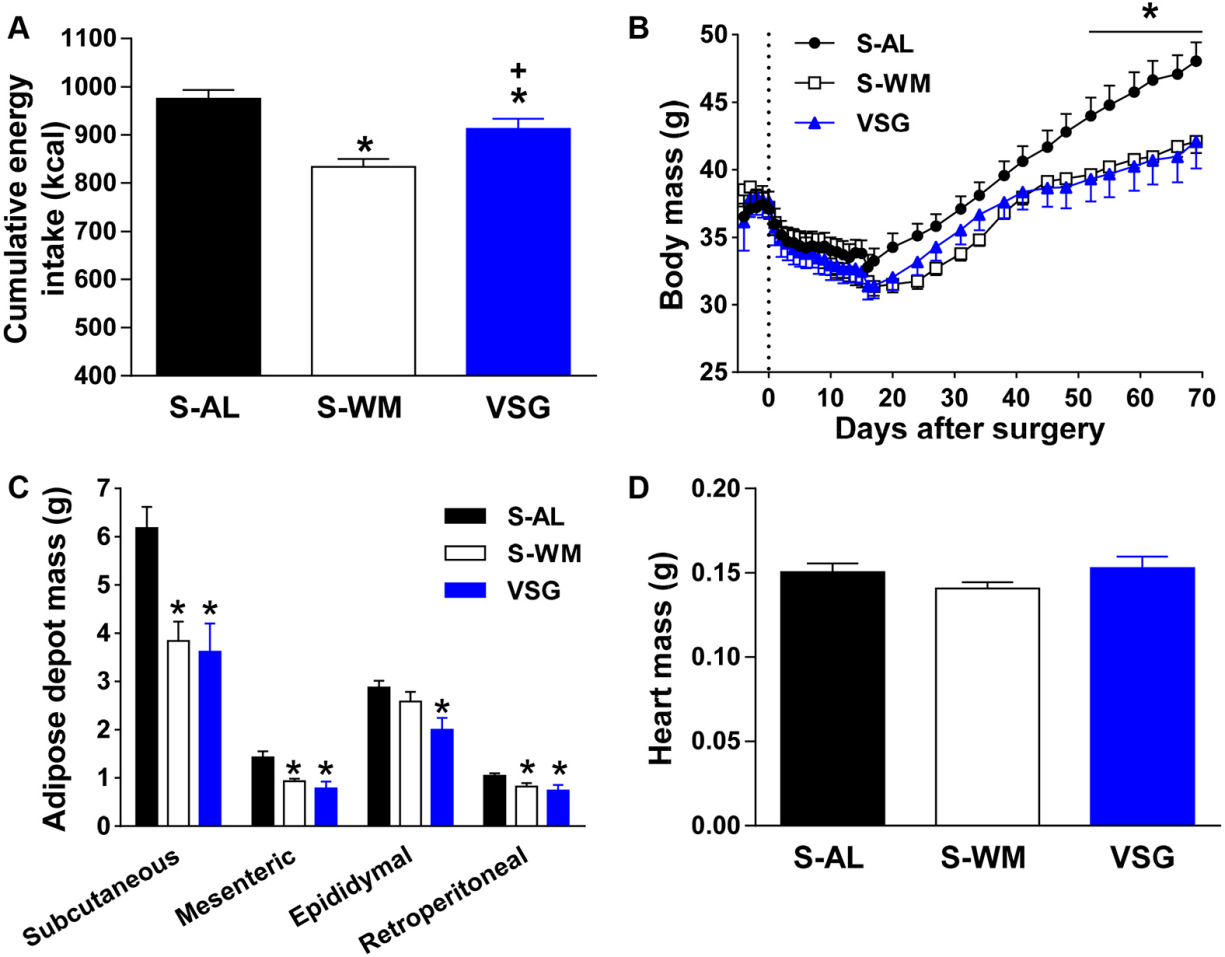
**VSG reduces body** **mass** **and energy intake.** The effect of VSG surgery on cumulative energy intake (A), body mass (B) white adipose tissue depot mass (C) and heart mass (D) in VSG-operated mice (VSG) and sham-operated *ad libitum*-fed or weight-matched mice (S-AL and S-WM, respectively). *P<0.05, compared with S-AL; ^+^*P*<0.05, compared with S-WM by two-factor ANOVA for body mass and by Student's *t*-test for all other variables. Data are expressed as mean±s.e.m.

Similar to our previous work (McGavigan et al., 2015), VSG surgery reduced cumulative energy intake, body mass and fat mass compared with sham-operated mice fed *ad libitum* (S-AL) (Fig. 1A-C; *P*<0.05). Body mass and white adipose depot mass (mesenteric, retroperitoneal, epididymal and subcutaneous) did not differ between VSG and sham-operated weight-matched (S-WM) controls (Fig. 1B,C). Therefore, we were able to assess the body mass-independent effect of VSG surgery on AP. Similar to previous studies in rats (Rodríguez et al., 2012a,b), heart mass did not significantly differ between groups (Fig. 1D).

**Fig. 2.**
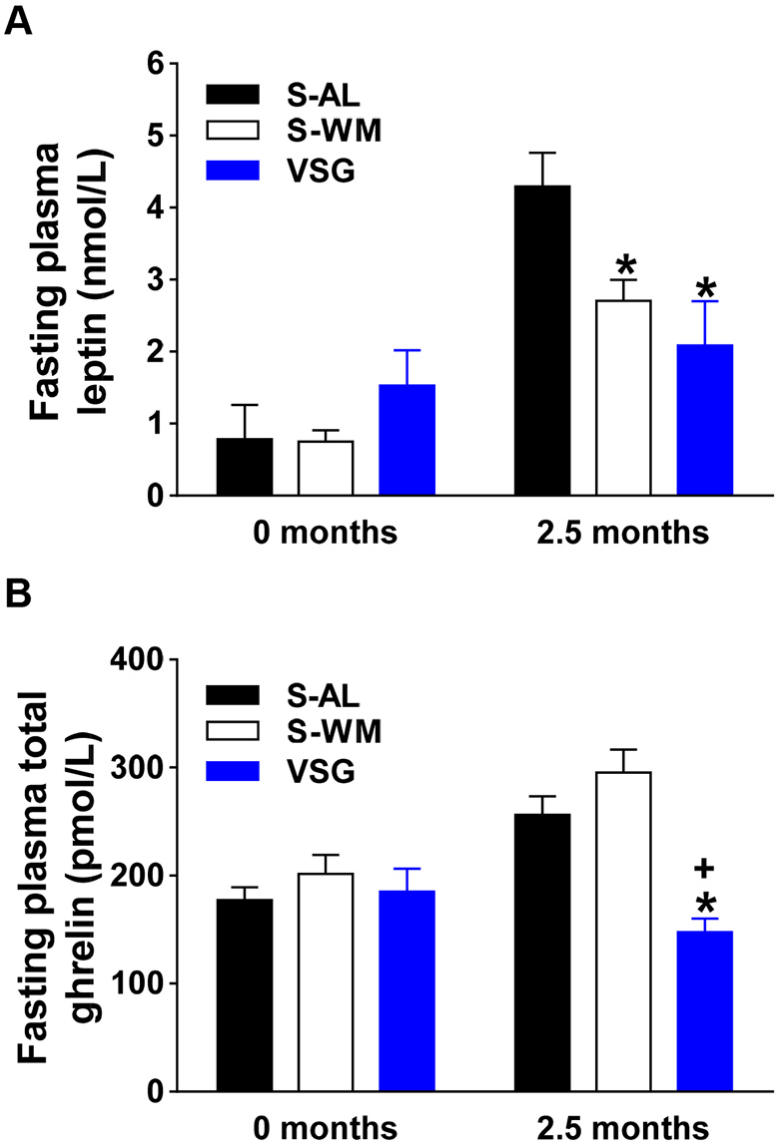
**VSG decreases fasting plasma leptin and ghrelin concentrations.** Fasting plasma leptin (A), and total ghrelin (B) concentrations at baseline and at 2.5 months after surgery in VSG-operated mice (VSG) and sham-operated *ad libitum*-fed or weight-matched mice (S-AL and S-WM, respectively). *P<0.05, compared with S-AL; ^+^*P*<0.05, compared with S-WM by two-factor ANOVA. Data are expressed as mean±s.e.m.

Leptin is an adipocyte-derived hormone that is often elevated in obesity and has been shown to contribute to obesity-associated increases in blood pressure (Simonds et al., 2014). As expected, VSG surgery significantly reduced circulating leptin levels compared with levels in S-AL mice at 2.5 months after surgery (Fig. 2A; *P*<0.05). However, there was no difference in circulating leptin concentrations between VSG and S-WM mice (Fig. 2A). Ghrelin is an orexigenic hormone produced by enteroendocrine cells located in the gastric mucosa that promotes weight gain and insulin resistance (Sun et al., 2006). Circulating ghrelin concentrations, which are decreased following VSG in humans and rodent models, have been suggested to contribute to the metabolic benefits of VSG surgery (Cummings et al., 2012; Mans et al., 2015). Consistent with previous reports (Cummings et al., 2012; Mans et al., 2015), our murine VSG model exhibited decreased fasting plasma total ghrelin concentrations compared with S-AL and S-WM controls (Fig. 2B; *P*<0.05).

### VSG surgery decreases AP

**Fig. 3.**
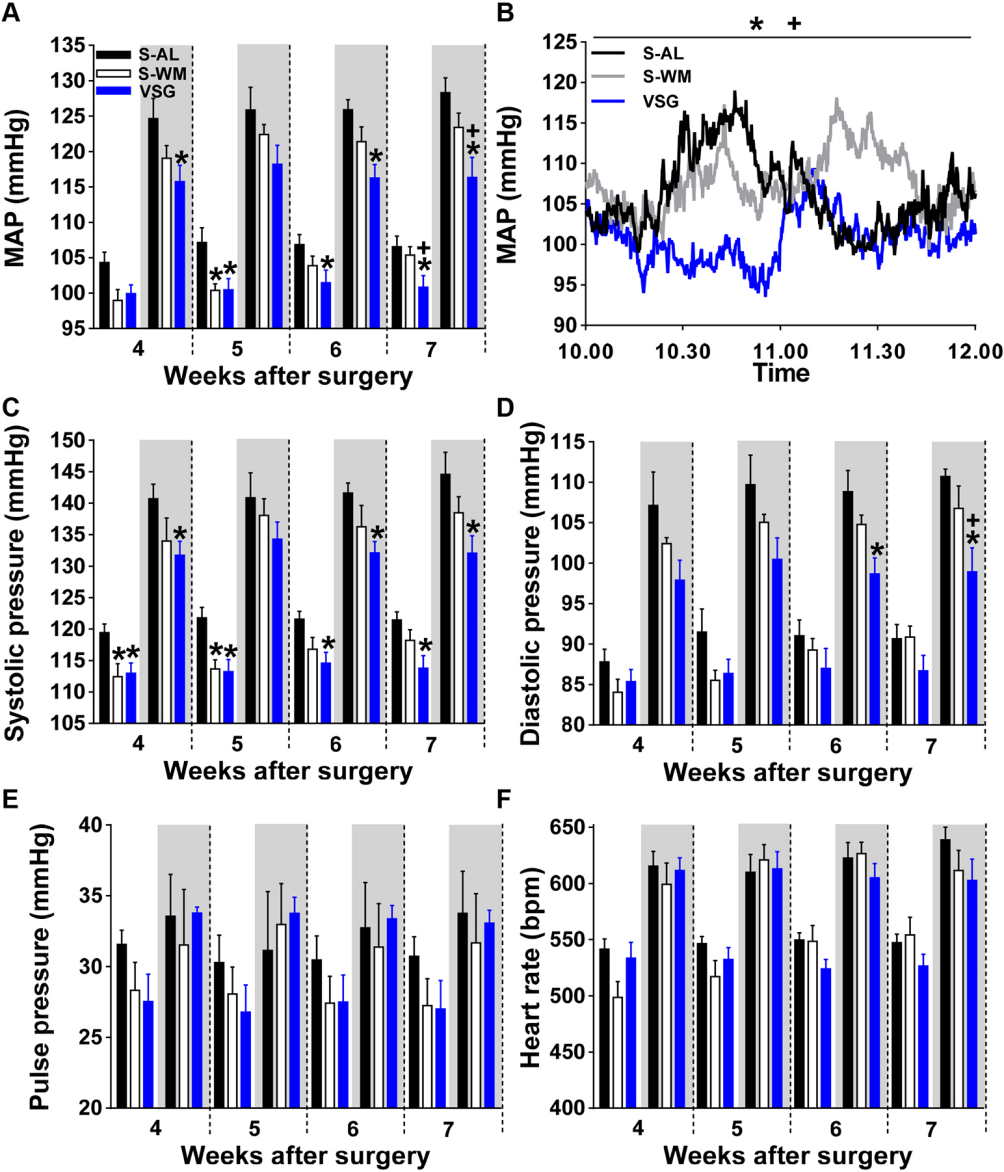
**VSG reduces mean arterial blood pressure.** (A) The effect of VSG surgery on MAP. (B) Representative trace of MAP during week 7 after surgery. The effect of VSG surgery on systolic pressure (C), diastolic pressure (D), pulse pressure (E) and on heart rate (F). VSG, VSG-operated mice; S-AL, sham-operated *ad libitum*-fed mice; S-WM, sham-operated weight-matched mice. *P<0.05, compared with S-AL; ^+^*P*<0.05, compared with S-WM by two-factor ANOVA. Shaded areas of graphs indicate measurements made during the dark phase and unshaded areas indicate measurements made during the light phase. Data are expressed as mean±s.e.m.

Mean arterial blood pressure (MAP) was reduced in S-WM relative to S-AL mice during the light phase at 5 weeks after surgery (Fig. 3A, *P*<0.05). In contrast, MAP was reduced in VSG-operated mice compared with S-AL animals during the light and/or dark phases during post-operative weeks 4-7 (Fig. 3A, *P*<0.05). MAP was lower in VSG compared with S-WM during both the light and dark cycles at 7 weeks after surgery, demonstrating a body mass-independent effect of VSG surgery to reduce MAP (*P*<0.05). Of note, body mass did not significantly differ between groups during weeks 4-6, emphasizing the body mass-independent reduction in MAP after VSG. A representative trace taken from week 7 after surgery is shown in Fig. 3B. These reductions in MAP are consistent with the human clinical literature which reports reductions of 5-10 mmHg in MAP after RYGB and VSG (Carson et al., 1994; Hinojosa et al., 2009; Seravalle et al., 2014). Our VSG model is also consistent with work done in a DIO rat model of VSG that reports similar reductions in MAP compared with *ad libitum*-fed control rats by tail-cuff plethysmography (Moncada et al., 2016; Rodríguez et al., 2012a).

Both S-WM and VSG-operated groups exhibited significantly lower systolic blood pressure compared with S-AL mice during the light cycle in weeks 4-5 after surgery (Fig. 3C, *P*<0.05). However, during weeks 6-7 after surgery, only VSG significantly lowered systolic blood pressure compared with S-AL (*P*<0.05). Furthermore, only VSG mice showed a significantly lowered systolic blood pressure compared with S-AL animals during the dark cycle, when activity levels and AP are highest (Fig. 3C, *P*<0.05). Diastolic blood pressure and pulse pressure did not differ between groups during the light cycle (Fig. 3D,E). However, diastolic blood pressure was decreased in VSG compared with S-AL and S-WM mice at 7 weeks after surgery during the dark cycle (Fig. 3D *P*<0.05). Heart rate did not differ between the groups (Fig. 3F, *P*<0.05).

The impact of VSG on MAP and systolic blood pressure was similar in the light and dark cycles, demonstrating that VSG-induced improvements in MAP occur independent of light cycle and activity level. However, the effect of VSG to lower diastolic blood pressure was only observed in the dark cycle, demonstrating that VSG only decreases diastolic blood pressure when activity and AP are high. This suggests that VSG-induced reductions in MAP during the light cycle are primarily due to a lowering in systolic blood pressure, whereas VSG-induced reductions in MAP during the dark cycle are due to a combination of reduced systolic and diastolic blood pressure. VSG-induced reductions in MAP, systolic blood pressure and diastolic blood pressure could be due to reduced sympathetic output, which has been observed after bariatric surgery in human patients, and points to a neurogenic mechanism (Guyenet, 2006; Seravalle et al., 2014).

### VSG reduces hypothalamic ER stress and inflammatory signaling independent of body weight

**Fig. 4.**
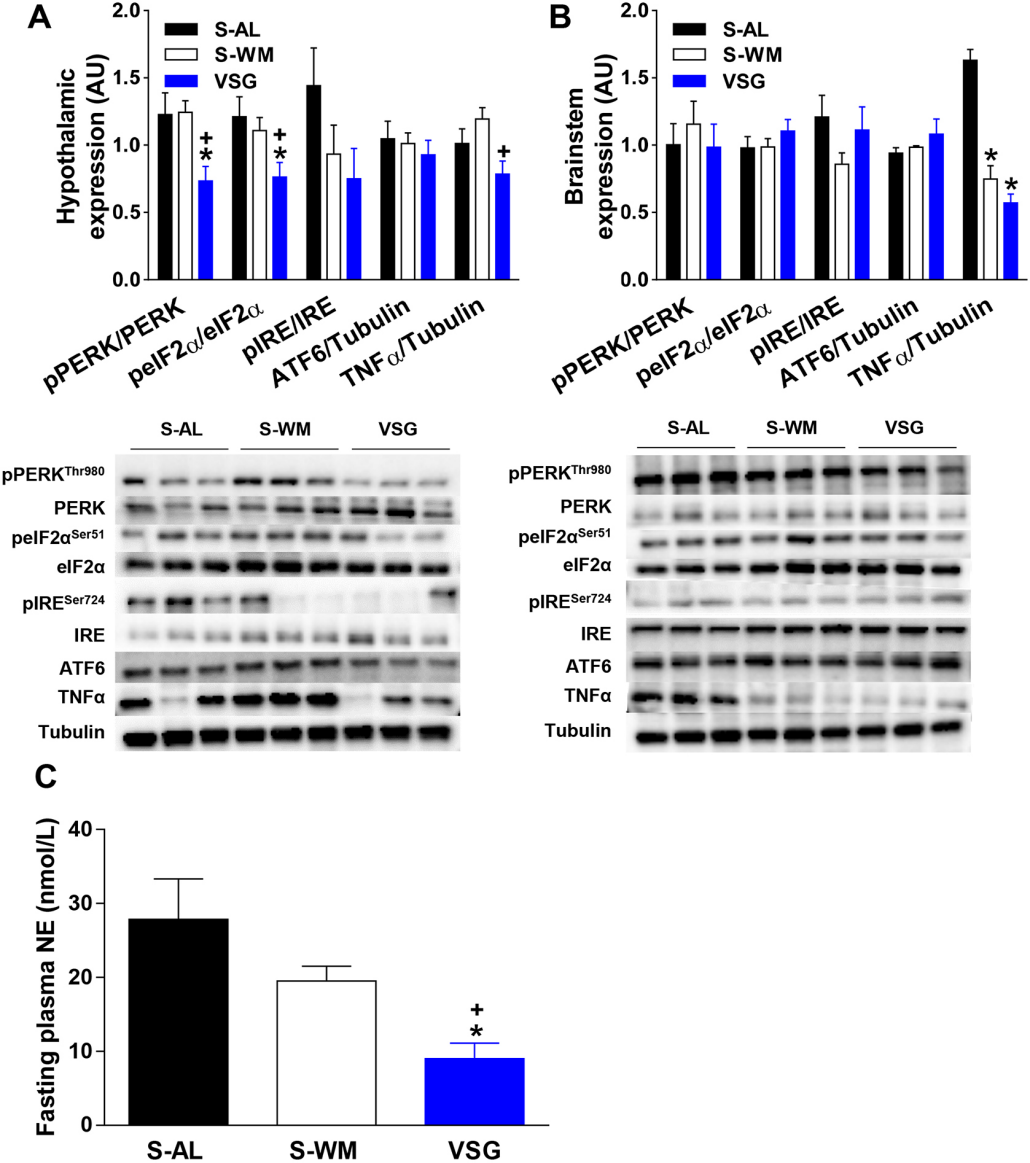
**VSG reduces hypothalamic ER stress, hypothalamic inflammatory signaling and circulating NE concentrations.** Representative immunoblots of pPERK^Thr980^, PERK, peIF2α^Ser51^, eIF2α, pIRE1α^Ser724^, IRE1α, ATF6, TNFα and tubulin (bottom) (*n*=3 per group). Results are quantified in densitometric units and pPERK^Thr980^ is expressed relative to PERK, peIF2α^Ser51^ relative to eIF2α, pIRE1α^Ser724^ relative to IRE1α and ATF6 and TNFα relative to tubulin (top) in hypothalamus (A) and brainstem (B) samples collected 2.5 months after surgery (*n*=6 per group). (C) Fasting plasma NE at 2.5 months after surgery. VSG, VSG-operated mice; S-AL, sham-operated *ad libitum*-fed mice; S-WM, sham-operated weight-matched mice. *P<0.05, compared with S-AL; ^+^*P*<0.05, compared with S-WM by Student's *t*-test. Data are expressed as mean±s.e.m.

It is well documented that obesity increases ER-stress signaling (Ron and Harding, 2012). Elevated ER-stress signaling in brain regions such as the hypothalamus and brainstem increases AP (Chao et al., 2013; Purkayastha et al., 2011b). Furthermore, obesity induced by a high-fat diet has been shown to elevate hypothalamic ER stress and AP (Purkayastha et al., 2011b). Therefore, we investigated the effect of VSG on all three pathways of ER-stress signaling in the hypothalamus and brainstem. VSG surgery reduced phosphorylation of hypothalamic PERK and its downstream mediator, eIF2α, compared with S-AL and S-WM mice (Fig. 4A; *P*<0.05). While IRE1α phosphorylation tended to be decreased in VSG-operated mice, this did not reach significance. Cleaved ATF6 protein expression did not differ between groups (Fig. 4A). Therefore, VSG decreases hypothalamic PERK-mediated ER-stress signaling independent of body mass.

All three ER-stress signaling pathways converge to activate NF-κB and thereby promote production of inflammatory mediators, such as TNFα. Hypothalamic inflammation has been implicated in the pathogenesis of hypertension (Harrison et al., 2011), with previous work reporting that an increase in hypothalamic TNFα raises AP (Purkayastha et al., 2011a). Therefore, we measured hypothalamic expression of TNFα and found a significant reduction in VSG-operated animals compared with S-WM (Fig. 4A; *P*<0.05). Although TNFα tended to be decreased after VSG compared with levels in S-AL mice, this did not reach significance. VSG-induced reductions in hypothalamic TNFα expression may be a result of decreased ER stress signaling and may also contribute to post-operative reductions in AP.

Interestingly, hypothalamic ER stress and inflammatory signaling did not significantly differ between S-AL and S-WM groups (Fig. 4A). Previous work has shown that obesity induced by high-fat diets increases hypothalamic inflammation and ER stress (Purkayastha et al., 2011a). However, we are not aware of a previous study that has differentiated between the effect of diet and body mass on hypothalamic ER stress and inflammatory signaling. Our data suggest that a high-fat diet increases hypothalamic ER stress and inflammation, which is at least partly independent of body mass.

ER-stress signaling in the brainstem did not differ between groups (Fig. 4B). TNFα was elevated in S-AL mice compared with S-WM and VSG groups, but did not differ between S-WM and VSG (Fig. 4B, *P*<0.05). These data suggest that increased body mass increases inflammatory signaling in the brainstem. Interestingly, inflammation in the brainstem has been implicated in the pathogenesis of hypertension in non-obese spontaneously hypertensive rats (Waki et al., 2011), suggesting that brainstem inflammation may have contributed to elevated AP in S-AL mice. Together, these data demonstrate that reductions in ER stress in the brainstem do not contribute to VSG-induced decreases in AP. Furthermore, these data demonstrate that the effect of VSG to lower ER stress and inflammation is not found throughout the brain.

Reductions in hypothalamic ER stress and inflammatory signaling have been shown to reduce AP by decreasing sympathetic outflow (Chao et al., 2013; Purkayastha et al., 2011a,b). Indeed, work in rodent models shows that increased ER stress and inflammatory signaling in the hypothalamus increases AP and sympathetic tone, and sympathetic suppression prevents the effect of hypothalamic ER stress and inflammation to increase AP (Purkayastha et al., 2011a,b). Furthermore, both RYGB and VSG surgery are associated with a decrease in sympathetic tone in humans (Curry et al., 2013; Seravalle et al., 2014), which has been suggested to contribute to the AP-lowering effect of bariatric surgery (Zhang et al., 2014). Therefore, we measured fasting plasma norepinephrine (NE) concentrations as an indicator of sympathetic activity. Consistent with the human clinical literature, VSG reduced circulating NE concentrations compared with levels in S-AL and S-WM mice (Fig. 4C, *P*<0.05). Therefore, VSG-induced reductions in hypothalamic ER stress may decrease AP by decreasing sympathetic tone.

### VSG surgery produces shifts in gut microbial populations

**Fig. 5.**
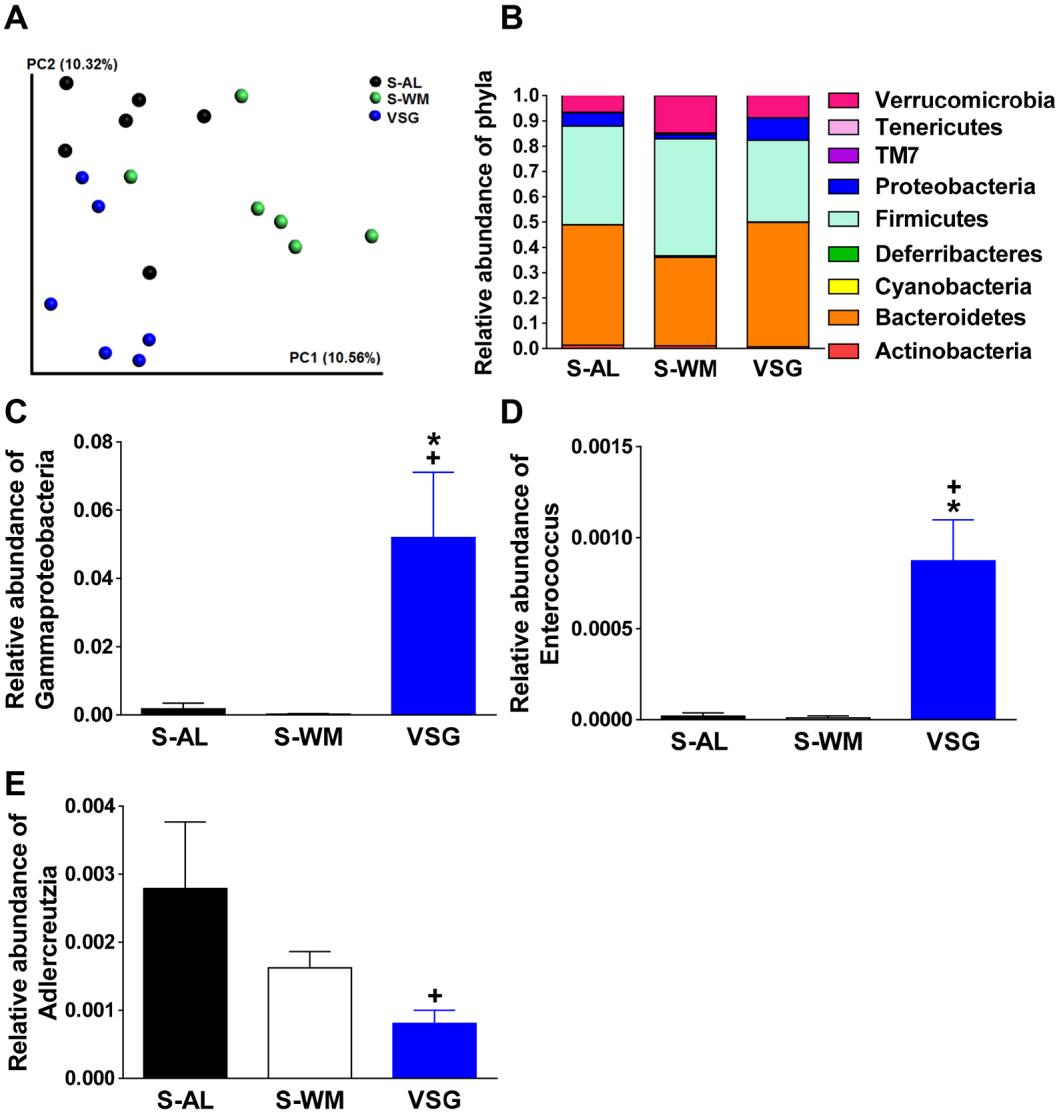
**VSG produces shifts in cecal microbial profiles.** Gut microbial profiles were determined in cecal content samples collected 2.5 months after surgery. (A) Principal coordinate analysis plots of unweighted UniFrac distances. (B) Relative abundance of bacterial phyla. Relative abundance of Gammaproteobacteria (C), *Enterococcus* (D), and *Adlercreutzia*. (E). Relative abundances are expressed as a percentage of total 16S rRNA gene sequences. VSG, VSG-operated mice; S-AL, sham-operated *ad libitum*-fed mice; S-WM, sham-operated weight-matched mice. *P<0.05, compared with S-AL; ^+^*P*<0.05, compared with S-WM. Data are expressed as mean±s.e.m.

Alterations in gut microbial populations have been suggested to contribute to the benefits produced by bariatric surgery, including VSG. The effect of bariatric surgery on the gut microbiome has been primarily characterized after RYGB, with few studies investigating the impact of VSG on gut microbial populations. Furthermore, the gut microbiota has been implicated in the modulation of brain inflammatory status (Wang and Kasper, 2014). Therefore, we assessed the impact of VSG on gut microbial populations by 16S rRNA gene sequencing. Principal coordinates analysis (PCoA) of the weighted and unweighted UniFrac distances between samples revealed differential clustering of VSG-operated mice compared with S-WM along PC1 and compared with S-AL groups along PC2 (Fig. 5A; *P*<0.05). Rarefaction analysis revealed no significant differences between groups in gut microbiome richness (α-diversity). No significant differences in community structure between groups were noted at the phylum level (Fig. 5B).

Similar to RYGB (Liou et al., 2013; Tremaroli et al., 2015), VSG resulted in significant elevations in the relative abundance of Gammaproteobacteria (Fig. 5C; *P*<0.05). Furthermore, VSG also increased the relative abundance of Enterobacteriales and Enterobacteriaceae, an order and family of Gammaproteobacteria, respectively (*P*<0.05). Interestingly, a previous study reports that alterations in Gammaproteobacteria are associated with central nervous system-dependent changes in exploratory behavior in mice (Bercik et al., 2011). Similar to our previous findings at 6.5 months after VSG surgery in mice (McGavigan et al., 2015), the family Enterococcaceae and genus *Enterococcus* were enriched in VSG compared with S-AL and S-WM (Fig. 5D; *P*<0.05). Similar to our previous findings, relative abundance of the family Coriobacteriaceae and genus *Adlercreutzia* were lower in VSG compared with S-WM (Fig. 5E; *P*<0.05). Interestingly, a reduction in the abundance of Coriobacteriaceae has been reported following RYGB in human patients (Zhang et al., 2009). Coriobacteriaceae have been suggested to promote cholesterol absorption (Claus et al., 2011; Martinez et al., 2009). VSG results in a body mass-independent reduction in circulating cholesterol concentrations (McGavigan et al., 2015). Thus, VSG-induced decreases in Coriobacteriaceae levels may contribute to the decrease in circulating cholesterol concentrations and thus improvements in cardiometabolic health. Overall, these data demonstrate that VSG-induced increases in Enterococcaceae, *Enterococcus*, Coriobactericeae and *Adlercreutzia* are durable gut microbial shifts after VSG.

## DISCUSSION

Here, we provide validation of a mouse model of VSG that recapitulates the effect of VSG to decrease AP in human patients. Our data show that there are both body mass-dependent and -independent mechanisms by which VSG reduces AP. This is consistent with previous work reporting reductions in AP prior to significant loss of body mass in humans (Ahmed et al., 2009). Furthermore, we demonstrate a body mass-independent reduction in hypothalamic ER stress and inflammatory signaling and body mass-independent shifts in gut microbial populations after VSG. Notably, this is the first rodent bariatric blood pressure study to control for body mass, the first study to employ radiotelemetry in a murine model of bariatric surgery and the first study to investigate a potential role for hypothalamic ER-stress signaling and the gut microbiome in mediating the reduction in AP after VSG.

Critically, this study is the first to control for body mass throughout the study, providing novel insight into the role of body mass in the effect of VSG to decrease AP. Previous studies have employed pair-feeding strategies to control for body mass. However, VSG increases energy expenditure, an effect that is present in our murine VSG model (McGavigan et al., 2015), making pair-feeding insufficient for controlling for body mass. Overall, we provide novel data showing that the VSG-induced reduction in AP is not totally dependent on body mass.

Endocrine factors regulate AP, and elevated circulating leptin concentrations are a common metabolic disturbance associated with obesity that have been implicated in the pathogenesis of hypertension (Simonds et al., 2014). Similar to previous studies, we show that VSG surgery decreases circulating leptin concentrations in comparison to sham-operated *ad libitum*-fed mice, but not weight-matched controls, suggesting that leptin does not contribute to the body mass-independent decreases in AP after VSG (Cummings et al., 2012). In line with this, previous work in a leptin receptor-deficient rat model reports reductions in AP after VSG despite the lack of a functional leptin receptor (Rodríguez et al., 2012b). Post-operative decreases in circulating ghrelin concentrations have been suggested to contribute to metabolic improvements after VSG surgery. In contrast, increased ghrelin signaling has been suggested to exert beneficial effects on AP regulation (Nagaya et al., 2001). Therefore, our data suggest that alterations in ghrelin do not contribute to decreased AP after VSG. This is consistent with previous work showing that ghrelin-knockout mice exhibit similar improvements in glucose regulation after VSG compared with wild-type mice (Chambers et al., 2013). Overall, post-operative reductions in circulating leptin and ghrelin concentrations do not appear to contribute to the body mass-independent reduction in AP after VSG surgery.

Enhanced ER stress and inflammatory signaling in the hypothalamus are important contributors to the development of obesity-associated hypertension (Purkayastha et al., 2011a,b). We present novel data demonstrating a body mass-independent decrease in hypothalamic ER stress and inflammatory signaling after VSG, which may contribute to post-operative decreases in AP; however, further studies are needed to demonstrate a causal relationship. Importantly, previous work has implicated the PERK-mediated arm of ER-stress signaling in the pathogenesis of hypertension (Young et al., 2012). Reductions in hypothalamic ER-stress and inflammatory signaling contribute to reductions in AP by reducing sympathetic outflow (Purkayastha et al., 2011a,b). Furthermore, RYGB and VSG surgery result in decreased sympathetic output in humans (Curry et al., 2013; Seravalle et al., 2014). Consistent with this, we find that our murine VSG model exhibits a body mass-independent reduction in circulating NE concentrations. Together, our data suggest that VSG-induced reductions in hypothalamic ER stress may decrease AP by decreasing sympathetic output.

The mechanism(s) by which the gut microbiota regulates AP are incompletely defined. Notably, numerous studies implicate the gut microbiota in the pathology of neuro-immune and neuro-psychiatric diseases, enforcing the concept of a microbiota-gut-brain axis and suggesting that the gut microbiome can be targeted for the treatment of diseases with a neurogenic basis (Wang and Kasper, 2014). Specifically, there are several reports of impacts made by the gut microbiome on metabolism and/or psychological outcomes in rodent models that are mechanistically dependent on interactions at the level of the brain (Cryan and Dinan, 2012; De Vadder et al., 2014; Foster and McVey Neufeld, 2013). Similar to our previous findings, we show that VSG surgery enriched Gammaproteobacteria and *Enterococcus* populations, and lowered *Adlercreutzia* populations. *Adlercreutzia* is a relatively newly identified genus of the phylum Actinobacteria (family Coriobacteriaceae). Interestingly, decreased *Adlercreutzia* was recently reported to be associated with relapsing remitting multiple sclerosis, an immune-mediated inflammatory disease of the central nervous system (Chen et al., 2016). Further work is required to demonstrate that the gut microbiota contribute to improvements in AP and central nervous system homeostasis after VSG.

In summary, we have demonstrated that VSG surgery reduces AP, but this is only partly dependent on body mass. We provide the first data to suggest that reductions in hypothalamic ER stress and inflammation may contribute to decreased AP after VSG. Further work is required to demonstrate a causal relationship between these post-operative changes and reduced AP after VSG. Of note, there are several other changes that occur after bariatric surgery in humans, which are replicated in our murine VSG model and may contribute. For example, we have previously reported increases in circulating glucagon-like peptide-1 (GLP-1) and bile acid concentrations in our murine VSG model and have demonstrated that these changes contribute to the glucoregulatory benefits of VSG (Garibay et al., 2016; McGavigan et al., 2015). Both GLP-1 and bile acids have been implicated in AP regulation and further investigation is required to determine their role in the AP-lowering effect of VSG (Drucker, 2016; Khurana et al., 2011). Ultimately, a complete understanding of the mechanisms by which bariatric surgery reduces AP will require a validated preclinical model that recapitulates the hallmark cardiometabolic improvements observed in humans. This work validates one such model and emphasizes the potential drug discovery opportunities associated with future studies in this model.

## MATERIALS AND METHODS

### Animals and diet

At 2 months of age, male C57BL/6J diet-induced obese mice were placed on a 60% energy from fat high-fat diet (HFD) (D12492, Research Diets). One week prior to surgery, mice were fasted for 6 h (08:00 h-14:00 h) and baseline fasting tail- blood samples were collected. At 4 months of age, mice underwent sham or VSG surgery (*n*=14) and were maintained on HFD throughout the study. Sham-operated mice were divided into two groups: *ad libitum* fed (S-AL) (*n*=11) or food restricted to match their body mass to VSG-operated mice (S-WM) (*n*=12). Food intake and body mass were measured twice per week, with the exception of S-WM animals, which received daily rations of food in order to match their body mass to the VSG-operated mice, as previously described (McGavigan et al., 2015). At 2.5 months after surgery, mice were fasted for 6 h (08:00 h-14:00 h) and a fasting tail-blood sample collected prior to euthanasia by an overdose of pentobarbital (200 mg/kg IP) for tissue collection. All animal procedures were approved by Cornell University Institutional Animal Care and Use Committee.

### VSG surgery

VSG and sham surgery were performed as previously described (McGavigan et al., 2015). Anesthesia was induced and maintained with isoflurane (1.5-5%). A laparotomy incision was made and the stomach was isolated outside the abdominal cavity. Approximately 70% of the stomach was removed, leaving a tubular remnant. Sham surgeries were performed by isolating the stomach and placing a simple continuous pattern of suture extending through the gastric wall and along both gastric walls in the same location as the VSG-operated mice.

### Telemeter implantation and recordings

Immediately following either sham or VSG surgery, mice were fitted with radiotelemetry probes (PA-C10; Data Sciences International), as previously described (Young et al., 2012). Briefly, the radiotelemeter catheter was implanted in the thoracic aorta via the left common carotid artery. The body of the probe was placed in a subcutaneous pocket on the right flank. Three light-phase recordings (10:00 h-12:00 h) were obtained weekly during weeks 4-7 after surgery in freely moving conscious mice in their home cages. In a subset of study mice (*n*=4-7), one dark-phase recording (24:00 h-02:00 h) was obtained weekly during weeks 4-7 after surgery.

### Measurement of circulating hormones

In a subset of 5-8 mice per group, plasma leptin and ghrelin levels were measured by sandwich electrochemiluminescence immunoassays (Meso Scale Discovery, Gaithersburg, MA, USA).

### Measurement of norepinephrine

In a subset of 7-10 mice per group, plasma NE was measured as previously described (Jia et al., 2011; Marce et al., 1995; Nirogi et al., 2013). Briefly, 50 µl of plasma was mixed with isotopical internal standard and protein was subsequently removed by adding 50 µl of 0.4 M perchloric acid. The clear supernatant was mixed with 100 µl of 1 M NaHCO_3_ and 200 µl dansyl chloride (1% in acetone) was added to the sample followed by heating at 65°C for 10 min. The sample was chilled on ice and dansylated norepinephrine was extracted with 200 µl ethyl acetate. The organic phase was evaporated under a nitrogen stream and resuspended in acetonitrile for UPLC-MS/MS injection. The assay was carried out on a Waters Xevo TQ MS ACQUITY UPLC system. Dansylated norepinephrine was separated on a Waters Acquity UPLC BEH Phenyl column (3 mm inner diameter×100 mm with 1.7 μm particles) and detected using positive electrospray ionization tandem mass spectrometry (ESI-MS/MS) under optimized multiple reaction monitoring (MRM) mode. The optimized transitions of norepinephrine and internal standard are 869.3→170.3 and 857.7→170.3, respectively.

### Immunoblotting

A subset of 6 mice per group were used for assessment of hypothalamic PERK, pPERK^Thr980^, eIF2α, peIF2α^Ser55^, IRE1α, pIRE1α^Ser724^, ATF6, TNFα and tubulin protein expression using standard methodology, as previously described (McGavigan et al., 2015). Antibodies against PERK, pPERK^Thr980^ and peIF2α^Ser55^ were from Cell Signaling (3179 and 3398, respectively). Antibodies against eIF2α, ATF6 and tubulin were from Santa Cruz (sc-133227, sc-22799, sc-23948, respectively) and TNFα, IRE1α and pIRE1α^Ser724^ antibodies were from Abcam (ab66579, ab37073, ab104157, respectively). All antibodies were used at a concentration of 1:1000 and pixel densities of immunoreactive bands were quantified using FluorChem 9900 (Alpha Innotech, CA).

### Gut microbial analysis

Gut microbial populations were analyzed in a separate cohort of S-AL, S-WM and VSG mice (*n*=6 per group). C57BL/6J mice were placed on 45% energy from fat high-fat diet at 2 months of age and maintained on this diet throughout the study. Mice underwent sham or VSG surgery at 4 months of age and cecal content samples were collected 2.5 months after surgery. Gut microbial populations were analyzed as previously described (McGavigan et al., 2015). Briefly, DNA was isolated from cecal contents using the MoBio PowerSoil DNA Isolation Kit (Carlsbad, CA). The V4 region of the 16S rRNA gene was PCR amplified using barcoded 515F and 806R primers and sequenced on an Illumina MiSeq at the Cornell Biotechnology Resource Center Genomics Facility. Beta-diversity was calculated using the unweighted and weighted UniFrac metrics on an operational taxonomic unit (OTU) table rarefied to 38,000 sequences per sample (Lozupone et al., 2007). Taxonomic groups that were differentially abundant between treatments were identified using the Galaxy version of LEfSe (Segata et al., 2011).

### Statistical analysis

Data are expressed as means±s.e.m. All statistical analyses were performed using GraphPad Prism v.6.00. Data were analyzed using ANOVA with Newman–Keuls *post hoc* analysis or by Student's *t*-test, as indicated. Differences were considered significant at *P*<0.05.
